# Stress Hyperglycemia Ratio as a Prognostic Marker in Diabetic Patients Hospitalized with COVID-19

**DOI:** 10.3390/idr14050073

**Published:** 2022-09-07

**Authors:** Mohamed Aon, Abdullah Alsaeedi, Azeez Alzafiri, Abdelrahman Al-Shammari, Sherif Taha, Omar Al-Shammari, Mahmoud Tawakul, Jarrah Alshammari, Naser Alherz, Monerah Alenezi, Meshari Eyadah, Mariam Aldhafeeri, Teflah Alharbi, Duaa Alshammari, Zaid Alenezi, Salem Aldouseri, Ebraheem Albazee, Mohamed M. Ibrahim, Ahmed H. Aoun

**Affiliations:** 1 Department of Internal Medicine, Faculty of Medicine, Cairo University, Giza 12613, Egypt; 2 Department of Internal Medicine, Jahra Hospital, Jahra 2675, Kuwait; 3 Jaber Al-Ahmad Military Hospital, Ministry of Defense, Kuwait City 46001, Kuwait; 4 Department of Pediatrics, Faculty of Medicine, Cairo University, Giza 12613, Egypt

**Keywords:** COVID-19, diabetes mellitus, hyperglycemia, SARS-CoV-2, stress hyperglycemia ratio

## Abstract

Evidence is conflicting about the diabetes characteristics associated with worse outcome among hospitalized COVID-19 patients. We aimed to assess the role of stress hyperglycemia ratio (SHR) as a prognostic marker among them. In our retrospective cohort study, patients were stratified according to SHR, admission glucose, and glycated hemoglobin tertiles. The primary outcome was a composite endpoint of invasive mechanical ventilation, intensive care unit admission, and in-hospital mortality. The study included 395 patients with a mean age of 59 years, and 50.1% were males. Patients in the third tertile of SHR developed more primary events, and the difference was significant compared to the first tertile (*p* = 0.038) and close to significance compared to the second tertile (*p* = 0.054). There was no significant difference in the outcomes across admission glucose and glycated hemoglobin tertiles. A higher SHR tertile was an independent risk factor for the primary outcome (OR, 1.364; 95% CI: 1.014–1.836; *p* = 0.040) after adjustment for other covariables. In hospitalized COVID-19 diabetic patients, SHR third tertile was significantly associated with worse outcome and death. SHR can be a better prognostic marker compared to admission glucose and glycated hemoglobin. A higher SHR was an independent risk factor for worse outcome and in-hospital mortality.

## 1. Introduction

The coronavirus disease (COVID-19) pandemic, which was caused by the severe acute respiratory syndrome coronavirus 2 (SARS-CoV-2), has spread rapidly to involve the whole world [1]. The clinical course of COVID-19 is highly variable with 14% and 5% of cases having severe and critical disease, respectively [2]. Epidemiologic data revealed that the presence of comorbidities such as diabetes mellitus (DM) is well-known to be associated with more severe COVID-19 and a worse outcome [3]. Overall, diabetic patients are at a higher risk of respiratory infections compared to non-diabetics [4]. Furthermore, DM was identified as a risk factor for morbidity and mortality among patients infected with coronaviruses, e.g., Middle East respiratory syndrome coronavirus (MERS-CoV) [5] and severe acute respiratory syndrome (SARS) [6]. Among COVID-19 patients, DM was the second most frequently reported comorbidity, and was associated with increasing severity and death [7]. A diabetes prevalence of 19% and 22.6% among hospitalized COVID-19 patients was reported in studies from China and USA, respectively [8] [9]. In Kuwait, diabetic patients represented 35% of hospitalized COVID-19 patients and DM was the most frequently reported comorbidity [10]. Therefore, diabetic patients are expected to constitute a significant number of hospitalized COVID-19 patients. The identification of DM phenotypes associated with poor prognosis is necessary for the best management of those vulnerable patients. Although many medical organizations have listed diabetic patients as a high-risk group for severe COVID-19, information regarding diabetes characteristics and phenotypes associated with disease severity is not enough [11,12]. A study from China revealed that poorly controlled blood glucose during hospitalization correlated with higher mortality [13]. A French study showed that higher admission glucose levels, but not glycated hemoglobin (HbA1c) levels, were associated with higher rates of invasive mechanical ventilation (IMV) and death [14,15]. A population-based study from England found that higher HbA1c levels were associated with increased COVID-19 mortality [16]. Higher glucose and HbA1c levels were reported among non-survivor hospitalized diabetic patients compared to survivors [17]. Other studies found that admission hyperglycemia was associated with increased COVID-19 mortality in non-diabetic patients [18,19]. Another study found that both higher admission glucose and higher peak glucose levels during corticosteroids treatment were associated with less successful extubation and higher mortality, irrespective of preexisting diabetes [20]. These conflicting results highlight the need for a more accurate glycemia metric that reflects both acute and chronic glycemic control in diabetic patients. The stress hyperglycemia ratio (SHR) is a better indicator of critical illness than absolute hyperglycemia because it controls for background hyperglycemia. It is calculated as admission glucose divided by the estimated average glucose (eAG) [21]. In our study, we aimed to investigate the association between SHR and the disease severity among diabetic patients hospitalized with COVID-19. We also investigated the association between other hyperglycemia metrics (e.g., admission glucose and HbA1c) and COVID-19 outcomes among diabetic patients hospitalized with COVID-19.

## 2. Materials and Methods

### 2.1. Study Design and Settings

Our study was a retrospective cohort study. The study included diabetic patients who were admitted with COVID-19 pneumonia between November 2020 and September 2021 to Jahra Hospital, Kuwait (the main district hospital). HbA1c and admission glucose were measured in venous blood samples using a G8 HPLC Analyzer (TOSOH bioscience Inc., San Francisco, CA, USA) and a DxC AU chemistry analyzer (Beckman Coulter Inc., Brea, CA, USA), respectively. Patients were stratified according to SHR, admission glucose, and HbA1c tertiles. All hospitalized patients were treated according to the national treatment protocol that included supportive care, thromboprophylaxis for all patients unless contraindicated, and corticosteroids for hypoxic patients (SO2 < 94% while breathing ambient air). Diabetic patients were treated with a basal and prandial insulin regimen targeting a glucose level of 7.8–10 mmol/L [22,23].

### 2.2. Inclusion and Exclusion Criteria

Diabetic patients were included in the study if they fulfilled all the following criteria: (1) hospitalization with COVID-19 pneumonia (clinical and/or radiological evidence of lower respiratory disease, e.g., shortness of breath, hypoxia, and/or lung involvement on chest x-ray); (2) the diagnosis of COVID-19 was confirmed by a positive polymerase chain reaction (PCR) for SARS-CoV-2; (3) HbA1c was measured on admission. Patients who were transferred to another hospital before the outcome was known or did not have an HbA1c measurement were excluded from the study. Pregnant females were excluded from the study. Patient demographics, clinical data, and admission laboratory parameters were obtained from the hospital records.

### 2.3. Operational Definitions

Diabetes was defined as HbA1c ≥ 6.5%, self-reporting of physician-diagnosed DM, or use of hypoglycemic drugs (oral or injectable). SHR and eAG were estimated using the following formulas: [21,24].

eAG (mmol/L) = (1.59 × HbA1c%) − 2.59(1)

SHR = admission glucose (mmol/L)/eAG (mmol/L)(2)

To define tertiles of SHR, we calculated the points that divide the ordered values of SHR into three parts. The same method was used to define tertiles of admission glucose and HbA1c.

### 2.4. Outcome Measures

The severity of COVID-19 was assessed using an ordinal scale consisting of the following categories: (1) not hospitalized and independent; (2) not hospitalized, but needs assistance; (3) hospitalized with no oxygen therapy; (4) hospitalized and required oxygen by mask or nasal prongs; (5) hospitalized and required high flow oxygen therapy (high-flow nasal cannula or noninvasive ventilation); (6) hospitalized and required IMV; (7) hospitalized and required IMV plus additional organ support, e.g., vasopressors, extracorporeal membrane oxygenation, and/or renal replacement therapy [25]. The admission scale and the worst ordinal scale during admission were recorded. Clinical deterioration was defined as an increase in the ordinal scale ≥ 2 steps. The primary outcome was a composite endpoint of intensive care unit (ICU) admission, the requirement of IMV, and 28-day in-hospital mortality. The secondary outcomes were clinical deterioration, the requirement of IMV, ICU admission, and 28-day in-hospital mortality.

### 2.5. Statistical Analysis

Validated data were tabulated, entered, and analyzed using Statistical Package for the Social Sciences (SPSS) version 22.0 (SPSS Inc., Chicago, IL, USA). Qualitative data were expressed as frequencies and percentages and comparisons between groups were performed using the Chi-square (χ^2^) test. Quantitative data were expressed as means and standard deviations (SDs) if data were normally distributed. The student *t*-test was used for comparisons of normally distributed quantitative variables. If the quantitative data were not normally distributed, they were expressed as medians and interquartile ranges. Differences between the study groups were compared using the Kruskal–Wallis test and post hoc analysis was done using Mann–Whitney U test. Logistic regression analysis was performed to detect the effect of SHR tertiles on the primary outcome. The effect was presented as odds ratio (OR) and 95% confidence interval (CI) after controlling for covariables. *p* value < 0.05 was considered statistically significant. Assuming an incidence of the main outcome of 16.7% and 33.3% in each group [26], 106 patients would be needed in each group to achieve a study power of 80% and a confidence level of 95%.

## 3. Results

### 3.1. Baseline Characteristics

**Figure 1:**
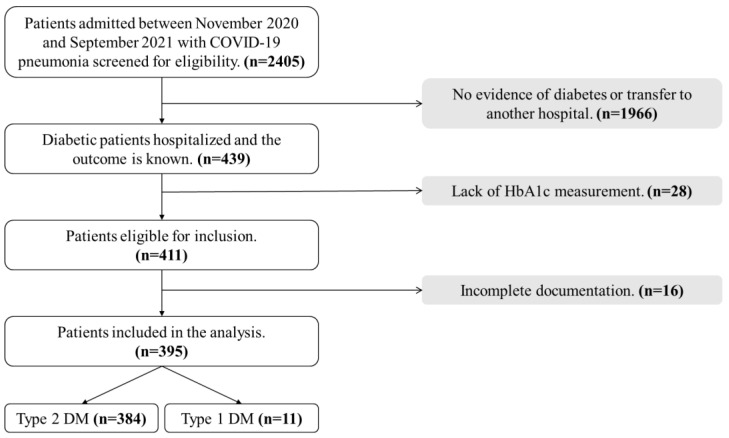
A flow chart demonstrating selection of the study cohort. Abbreviations: COVID-19, coronavirus disease 2019; DM, diabetes mellitus; HbA1c, glycated hemoglobin.

After application of the inclusion and exclusion criteria, our study included 395 diabetic patients hospitalized with confirmed COVID-19 pneumonia and most of them (97.2%) had type 2 DM, as demonstrated in Figure 1.

The mean age of patients in our cohort was 59.4 ± 13.3 years and 50.1% of patients were males. The most prevalent comorbidity was hypertension (55.2%), followed by atherosclerotic cardiovascular disease (41.8%). Table 1 demonstrates the baseline demographic, clinical, and laboratory characteristics of the whole cohort and their profiles according to SHR tertiles. Patients had a median admission glucose of 12.2 mmol/L and a median HbA1c of 8.6%. Patients in the third SHR tertile tended to have a lower lymphocytic count, lower hemoglobin, and higher admission glucose compared to the other tertiles.

### 3.2. Association between SHR and Outcomes

As demonstrated in Table 2, most of the patients in our cohort (84.6%) required oxygen therapy on admission. About 30% of the patients had the primary outcome in the whole cohort, 26.3% deteriorated clinically, 25.8% required IMV, and 17.5% died within 28 days of hospitalization. There was an upward trend in all primary and secondary outcomes with increasing SHR, most notably in the third tertile. When compared with the first tertile, the third tertile patients developed significantly more primary events and required ICU admission (*p* = 0.038). Although the incidence of clinical deterioration, IMV, and death were higher in the third tertile, the difference between first and third tertiles was not statistically significant. When the third tertile patients were compared with the second tertile, we found marginally significant higher primary event and ICU admission (*p* = 0.054). We also found a higher incidence of clinical deterioration, IMV, and death, which was not statistically significant.

### 3.3. Association between the Other Glycemia Metrics and Outcomes

When patients were stratified according to admission glucose tertiles, the difference in the primary and secondary outcomes across groups was not significant. Patients in the third tertile of admission glucose had a higher incidence of the primary outcome, but the difference did not reach statistical significance (*p* = 0.376). Likewise, when patients were stratified according to HbA1c tertiles, there was no significant difference in the primary or secondary outcomes across HbA1c tertiles as shown in Table 3.

### 3.4. Risk Factors for the Primary Outcome

**Figure 2:**
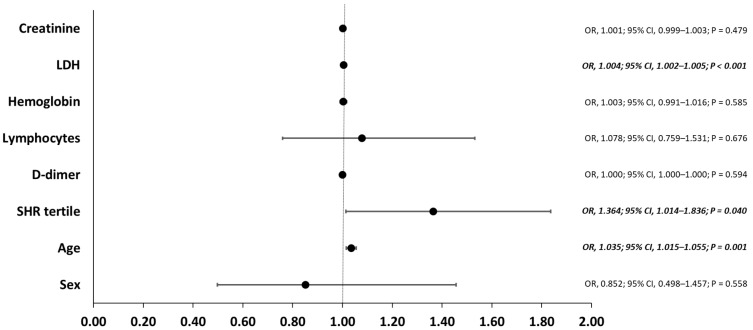
Risk factors for the primary outcome. For each variable, the black dot represent the odds ratio, and the horizontal line represent the 95% confidence interval. Abbreviations: CI, confidence interval; LDH, lactate dehydrogenase; OR, odds ratio; SHR, stress hyperglycemia ratio.

Logistic regression analysis was performed to identify the potential risk factors associated with the primary outcome. Candidate variables for the model were selected based on the analysis of variables across SHR tertiles and the risk factors previously described in COVID-19 patients. Our logistic regression model identified older age (OR, 1.035; 95% CI, 1.015–1.055; *p* = 0.001), higher SHR tertile (OR, 1.364; 95% CI, 1.014–1.836; *p* = 0.040), and elevated lactate dehydrogenase levels (OR, 1.004; 95% CI, 1.002–1.005; *p* < 0.001) as independent risk factors for the primary outcome after adjustment for other covariables as shown in Figure 2.

## 4. Discussion

The main finding in our study was the significant association between the highest (3rd) SHR tertile and the primary outcome (ICU admission, IMV, and in-hospital mortality) in diabetic patients. Although DM does not seem to increase the risk of acquiring COVID-19 infections, it is associated with a more severe disease and a worse prognosis [27]. Hyperglycemia modulates the hosts’ inflammatory and immune reactions, leading to a mixture of dysregulated immunity and maladjusted inflammatory response and consequently poorer outcomes. Likewise, COVID-19 predisposes infected patients to hyperglycemia, diabetic ketoacidosis, and new-onset diabetes [28]. This can lead to an endless loop of worsening hyperglycemia and COVID-19 infection [29].

A possible link between diabetes, inflammation, and COVID-19 could be the angiotensin-converting enzyme 2 (ACE2) and the dipeptidyl peptidase-4 (DPP4) receptors, which are expressed by pulmonary cells and can act as receptors for coronaviruses and, at the same time, are expressed in extrapulmonary tissues, playing an important role in the regulation of metabolic and inflammatory homeostasis [30].

Previous studies have explored diabetes characteristics associated with COVID-19 severity, but the results were contradictory. Holman et. al. identified an association between HbA1c and COVID-19 deaths in both types of diabetes (HbA1c ≥ 10% in type 1 DM and ≥7.6% in type 2 DM) [16]. Another study found that diabetic patients with HbA1c ≥ 7.5 had a higher COVID-19-related mortality [31]. On the contrary, other studies could not find an association between HbA1c levels and COVID-19 outcomes [32]. The CORONADO study demonstrated that HbA1c was not associated with mortality or requirement of IMV [14]. In our study, no significantly higher incidence of any of the primary or the secondary events was noticed across the HbA1c tertiles.

When we compared patients according to the admission glucose tertiles, the difference in primary and secondary outcomes across groups was not significant. The available evidence on the relation between acute hyperglycemia and COVID-19 includes different populations in different settings and uses different cutoff levels. Some studies linked higher glucose levels in diabetic patients with worse COVID-19 outcomes. Zhu et al. found that well-controlled blood glucose (≤10 mmol/L) was associated with lower mortality compared to poorly controlled blood glucose (>10 mmol/L) during hospitalization [13]. The CORONADO study demonstrated that admission glucose, compared to a reference value of 5.55 mmol/L, was associated with death and IMV. However, admission hyperglycemia was no longer associated with severity after adjustment for other laboratory covariates on admission [14,15]. A study from KSA found that neither blood glucose nor HbA1c affected the outcome in diabetic patients hospitalized with COVID-19 [33]. The association between acute hyperglycemia and worse COVID-19 outcome is not limited to diabetic patients. Wang et al. found that fasting glucose ≥7 mmol/L is an independent risk factor for mortality in COVID-19 patients without previous diabetes [18]. Wu et al. demonstrated that initial hyperglycemia was associated with progression to critical disease and death in non-diabetic patients [19]. Accordingly, it is unclear if admission hyperglycemia itself is a risk factor for worse COVID-19 prognosis or rather a marker of severity. Stress hyperglycemia may occur with any critical illness due to neurohormonal and inflammatory dysregulation [21]. Stress hyperglycemia aims to provide fuel for the brain and the immune system during periods of critical illness to improve survival [34,35]. Despite its being a defensive mechanism, stress hyperglycemia can lead to adverse effects due to the stimulation of oxidative stress and endothelial dysfunction [36]. The hypothesis that background hyperglycemia protects against the harmful effects of stress hyperglycemia due to down-regulation of glucose transporters may explain why COVID-19 patients with new hyperglycemia have poorer outcomes than patients with known diabetes [37].

Among diabetic patients, it was suggested that SHR, reflecting both admission glucose and HbA1c, represents the true stress hyperglycemia because in diabetic patients, absolute hyperglycemia may be a marker of long-term poor control rather than true stress hyperglycemia [38].

SHR controls for background glycemia and is more strongly associated with critical illness compared to absolute hyperglycemia in the presence of diabetes. This is similar to the superiority of body mass index over body weight as a prognosticator measurement [21]. Furthermore, in non-diabetic patients, hyperglycemia may indicate either stress hyperglycemia or undiagnosed DM. SHR is a valuable tool for evaluation in this situation [35].

In the present study, we hypothesized that SHR will adjust the influence of chronic hyperglycemia on COVID-19 severity. To the best of our knowledge, the relation between SHR and COVID-19 was explored in a single study on a limited number of patients. In the study by Ramon et al., 91 type 2 diabetic patients hospitalized with COVID-19 were stratified according to SHR tertiles (about 30 patients in each tertile) and compared in relation to a composite outcome of mortality, ICU admission, and IMV [26].

In the current study, we found a significant association of SHR third tertile, but not admission glucose or HbA1c, with IMV, ICU admission, and mortality. The third tertile patients represented patients with true stress hyperglycemia where the admission glucose was higher than the expected level according to HbA1c (eAG). Similar to our findings, Ramon et al. did not find an association between admission glucose tertiles or HbA1c tertiles and the primary outcome, but they did find an association with SHR tertiles. When they compared the primary outcome across SHR tertiles, third tertile (≥1.22) was associated with more primary events (*p* = 0.012) [26].

Furthermore, we demonstrated that a higher SHR tertile was an independent risk factor for the primary outcome after adjustment for other covariates. In accordance with our results, Ramon et al. identified SHR third tertile as an independent predictor of the primary outcome after adjustment for other covariables. They also reported a U-shaped association between mortality and SHR tertiles. The mortality was higher in the first and third tertiles compared to the second tertile (*p* = 0.045 and 0.064, respectively). They assumed that patients in the first tertile could have a state of relative hypoglycemia that led to higher mortality [26]. In our work, patients in the first tertile did not have a higher incidence of any of the primary or secondary events compared to the second or third tertile. Although their median admission glucose was less than the eAG, nearly all of them (98.5%) were euglycemic.

In our study, we did not use dichotomized SHR above or below specific numbers and preferred to compare SHR tertiles. In the original paper describing SHR, mortality increased in the fourth and fifth quintiles, corresponding to an average SHR of 1.14 and 1.38, respectively [21]. Previous studies in non-COVID-19 patients found that an SHR cutoff level of 1.14 or 1.38 has a prognostic value [35,39,40]. However, these cutoff levels were not validated and an SHR cutoff level remains inadequately defined.

Some points of strength need to be highlighted in the current work. To the best of our knowledge, this study is the first from the MENA region and the second worldwide to investigate the association between SHR and COVID-19 outcome. As mentioned above, a study from Spain investigated this association in a limited number of patients [26]. Another point of strength in the study is that all patients in our cohort were confirmed to have COVID-19 by PCR testing and all patients were treated according to the same treatment protocol. Additionally, samples of glucose and HbA1c were analyzed in the same standard laboratory to avoid any bias between different laboratories. Finally, admission glucose was measured, not glucose during hospitalization, which is more liable to variations and drug effects.

The current study has some limitations. First, it is a single-center study with an observational retrospective nature making it susceptible to the shortcomings of observational studies. Second, our study lacked data on subjects without diabetes, where HbA1c was not measured and subjects were not hospitalized; thus, our data cannot be generalized to all COVID-19 patients. Finally, we did not collect data on the detailed history of antidiabetic medications. However, all patients were treated with insulin during hospitalization.

## 5. Conclusions

In conclusion, our results demonstrated an association between higher SHR and worse COVID-19 mortality and outcomes. Our results suggested that SHR could act as a better prognostic marker compared to admission glucose and HbA1c. Adding SHR to other prognostic biomarkers in the presence of DM will help early identification of patients at risk of worse outcome. HbA1c and glucose are widely available, making SHR a simple personalized approach to patient management. Further studies are needed to evaluate COVID-19 patients based on their glycemic status and hyperglycemia metrics, rather than diabetes history. Such studies can guide better understanding of the clinical course of the disease and provide a personalized approach for management. In addition, studies examining the effects of glucose-lowering interventions are urgently needed to optimize the management of the disease. Until further results are available, we advise that diabetic patients should get vaccinated, abide by COVID-19 precautions, and maintain good glycemic control as a rule. If hospitalized, elevated SHR in diabetic patients should alarm healthcare workers as a prognostic marker of possible deterioration.

## Figures and Tables

**Table 1:** Demographics, comorbidities, and laboratory parameters for the patients according to SHR tertiles.

	Total (n = 395)	SHR ≤ 0.89 (1st Tertile, n = 131)	SHR 0.90–1.22 (2nd Tertile, n = 137)	SHR ≥ 1.23 (3rd Tertile, n = 127)	*p*-Value	Normal Range
Age (y), mean ± SD	59.37 ± 13.33	60.17 ± 13.14	57.72 ± 11.6	60.32 ± 15.08	0.200	
Sex, n (%)						
Male Female	198 (50.1) 197 (49.9)	63 (48.1) 68 (51.9)	72 (52.6) 65 (47.4)	63 (49.6) 64 (50.4)	0.758	
Comorbidity, n (%)						
Hypertension Renal disease ASCVD ^a^ Lung disease Cancer	218 (55.2) 57 (14.4) 165 (41.8) 40 (10.1) 4 (1)	84 (64.1) 16 (12.2) 49 (37.4) 13 (9.9) 0 (0)	66 (48.2) 14 (10.2) 55 (40.1) 12 (8.8) 2 (1.5)	68 (53.5) 27 (21.3) 61 (48) 15 (11.8) 2 (1.6)	0.029 0.026 0.200 0.711 0.365	
WBCs	7 (5–9.5)	6.5 (5–8.3)	6.9 (4.8–8.7)	7.6 (5.1–10.67)	0.196	4–10 × 10^9^/L
Neutrophils	5.1 (3.6–7.5)	4.4 (3.6–6.7)	5.2 (3.5–7.2)	5.75 (3.52–8.37)	0.167	2–7 × 10^9^/L
Lymphocytes	1.1 (0.7–1.5)	1.2 (0.7–1.5)	1 (0.7–1.6)	0.9 (0.6–1.4)	0.039	1–3 × 10^9^/L
Hemoglobin	126 (113–139)	129 (115–140)	130 (114–140)	124 (106.2–136.8)	0.018	130–170 g/L
Platelets	221 (172–275)	215 (175–265)	221 (177–287)	215.5 (167–270)	0.649	150–410 × 10^9^/L
Creatinine	88 (68–116.79)	82 (65–106.5)	87 (63–107)	89 (70–139)	0.220	57–113 mol/L
Albumin	28.1 (25.6–30.8)	28.6 (25.6–31.4)	27.9 (26.1–30.7)	27.4 (24.7–30.8)	0.250	35–55 g/L
ALT	29 (20–46)	29.5 (20.25–45)	28 (20–46)	27 (19–47)	0.804	8–41 IU/L
AST	37 (28–55)	38.5 (28–53)	35 (28–63)	32 (25–55)	0.203	10–40 IU/L
Ferritin	488.5 (240.6–852)	428 (221.6–893)	483 (211.1–863.2)	483 (257.9–885)	0.415	34–310 ng/ml
LDH	307 (237–383)	295 (228–371)	309.5 (246.3–399.5)	291.5 (220–397)	0.603	95–200 IU/L
D-dimer	342 (221–616.6)	332.5 (187–651.5)	286.7 (193.5–589.5)	345 (243.5–665)	0.185	<232 ng/ml
Admission glucose	12.2 (8.7–16.5)	7.7 (6.4–10.2)	11.5 (9.35–16)	17.5 (14.3–21)	<0.001	4–7 mmol/L
HbA1c	8.6 (7.1–10.6)	8.4 (7–10.5)	8.4 (7.1–10.75)	8.8 (7–10.4)	0.957	<6.5%
eAG	11.1 (8.7–14.3)	10.8 (8.6–14.1)	10.9 (8.7–14.6)	11.4 (8.6–14)	0.885	

^a^ Includes coronary heart disease, peripheral arterial disease, and stroke. Abbreviations: ALT, alanine aminotransferase; ASCVD, atherosclerotic cardiovascular disease; AST, aspartate aminotransferase; eAG, estimated average glucose; HbA1c, glycated hemoglobin; LDH, lactate dehydrogenase; SHR, stress hyperglycemia ratio; SD, standard deviation; WBCs, white blood cells; y, years. Note: All variables were expressed as median and interquartile range unless stated otherwise.

**Table 2:** The primary and secondary outcomes according to SHR tertiles.

	Total (n = 395)	SHR 1st Tertile	SHR 2nd Tertile	SHR 3rd Tertile	*p* Value ^a^	*p* Value ^b^
Admission scale ^c^ 3 4 5	61 (15.4) 329 (83.3) 5 (1.3)	21 (16) 107 (81.7) 3 (2.3)	20 (14.6) 115 (83.9) 2 (1.5)	20 (15.7) 107 (84.3) 0 (0)		
Primary outcome ^d^	118 (29.9)	34 (26)	37 (27)	47 (37)	0.038	0.054
Clinical deterioration ^e^	104 (26.3)	30 (22.9)	34 (24.8)	40 (31.5)	0.121	0.227
IMV	102 (25.8)	30 (22.9)	32 (23.4)	40 (31.5)	0.121	0.138
ICU	118 (29.9)	34 (26)	37 (27)	47 (37)	0.038	0.054
In-hospital mortality	69 (17.5)	20 (15.3)	22 (16.1)	27 (21.3)	0.212	0.227

^a^*p* value when 3rd tertile was compared to 1st tertile. ^b^ *p* value when 3rd tertile was compared to 2nd tertile. ^c^ Grades of the ordinal scale: [3] hospitalized with no oxygen therapy; [4] hospitalized and required oxygen by mask or nasal prongs; [5] hospitalized and required high flow oxygen therapy (HFNC or NIV). ^d^ The primary outcome was a composite endpoint of ICU admission, IMV, and 28-day in-hospital mortality. ^e^ Clinical deterioration was defined as an increase in the admission ordinal scale ≥ 2 steps. Abbreviations: HFNC, high-flow nasal cannula; ICU, intensive care unit; IMV, invasive mechanical ventilation; NIV, noninvasive ventilation; SHR, stress hyperglycemia ratio. Note: All variables were expressed as frequencies and (%).

**Table 3:** The primary and secondary outcomes according to admission glucose and HbA1c tertiles.

Outcomes	Admission Glucose ≤ 9.60 mmol/L (1st Tertile, n = 134)	Admission Glucose 9.61–14.90 mmol/L (2nd Tertile, n = 130)	Admission Glucose ≥ 14.91 mmol/L (3rd Tertile, n = 131)	*p*-Value
Primary outcome ^a^	36 (26.9)	37 (28.5)	45 (34.4)	0.376
Clinical deterioration ^b^	31 (23.1)	36 (27.7)	37 (28.2)	0.584
IMV	31 (23.1)	34 (26.2)	37 (28.2)	0.633
ICU	36 (26.9)	37 (28.5)	45 (34.4)	0.376
In-hospital mortality	19 (14.2)	25 (19.2)	25 (19.1)	0.467
	HbA1c ≤ 7.5% (1st tertile, n = 137)	HbA1c 7.6–9.9% (2nd tertile, 127)	HbA1c ≥ 10% (3rd tertile, 131)	
Primary outcome ^a^	40 (29.2)	41 (32.3)	37 (28.2)	0.760
Clinical deterioration ^b^	36 (26.3)	37 (29.1)	31 (23.7)	0.608
IMV	35 (25.5)	36 (28.3)	31 (23.7)	0.689
ICU	40 (29.2)	41 (32.3)	37 (28.2)	0.760
In-hospital mortality	26 (19)	21 (16.5)	22 (16.8)	0.846

^a^ The primary outcome was a composite endpoint of ICU admission, IMV, and 28-day in-hospital mortality. ^b^ Clinical deterioration was defined as an increase in the admission ordinal scale ≥ 2 steps. Abbreviations: HbA1c, glycated hemoglobin; ICU, intensive care unit; IMV, invasive mechanical ventilation. Note: All variables were expressed as frequencies and (%).

## Data Availability

The data supporting our findings are available from the corresponding author upon reasonable request and after approval from our IRB.
